# Interconnected PolymerS TeChnology (IPSTiC): An Effective Approach for the Modulation of 5α-Reductase Activity in Hair Loss Conditions

**DOI:** 10.3390/jfb9030044

**Published:** 2018-07-12

**Authors:** Ortensia Ilaria Parisi, Luca Scrivano, Fabio Amone, Rocco Malivindi, Mariarosa Ruffo, Anna Francesca Vattimo, Vincenzo Pezzi, Francesco Puoci

**Affiliations:** 1 Department of Pharmacy, Health and Nutritional Sciences, University of Calabria, 87036 Rende (CS), Italy; ortensiailaria.parisi@unical.it (O.I.P.); luca.scrivano@unical.it (L.S.); rocco.malivindi@unical.it (R.M.); mariarosaruffo8@gmail.com (M.R.); v.pezzi@unical.it (V.P.); 2 Macrofarm s.r.l., c/o Department of Pharmacy, Health and Nutrition Sciences, University of Calabria, 87036 Rende (CS), Italy; amonefabio@gmail.com (F.A.); vattimoanna@yahoo.it (A.F.V.)

**Keywords:** hair loss, androgenetic alopecia (AGA), 5α-reductase, polymeric blend, hyaluronic acid, soybean proteins, *Origanum vulgare* leaf extract, *Camellia Sinensis* leaf extract, *Capsicum Annuum* fruit extract, EPISKIN model

## Abstract

Hair loss represents a condition that adversely affects the social life of patients. The most common cause is androgenetic alopecia (AGA), which is a genetically determined progressive hair-loss condition involving 5α-reductase. In this study, a novel anti-baldness agent based on Interconnected PolymerS TeChnology (IPSTiC), which is an effective strategy for the delivery of bioactive molecules, was developed. This product (IPSTiC patch hair) is based on a polymeric blend consisting of high molecular weight hyaluronic acid and soybean proteins and is able to improve efficacy and stability of bioactive ingredients such as *Origanum vulgare* leaf extract, *Camellia Sinensis* leaf extract, and *Capsicum Annuum* fruit extract. The efficacy of the developed anti-baldness agent was investigated by performing several tests including NO radical and 5α-reductase inhibition assays, stability studies under different conditions, and in vitro diffusion studies using Franz cells. The biocompatibility of IPSTiC patch hair was also evaluated by in vitro analysis of the pro-sensitising potential and EPISKIN model. The obtained results confirmed both the efficacy and safety of IPSTiC patch hair supporting the potential use of this product in the topical treatment of AGA.

## 1. Introduction

Hair loss represents a harmful condition that adversely affects the quality of social life of patients. The main causes of hair loss include telogen effluvium, nutrition, endocrine imbalances, drugs, infections, special diseases, malignancy, stress, and environmental factors. In this context, the most common one is represented by androgenetic alopecia (AGA), which is a genetically determined hair-loss hereditary condition affecting a large number of both men and women [1,2]. This pathological condition involves an altered hair cycle with a progressive miniaturization of the hair follicle, which is smaller as a result.

The hair follicle is an epidermal structure, which undergoes repetitive cycles consisting of four main phases such as anagen (active growth), catagen (apoptosis-driven involution), exogen (hair shedding), and telogen (relative quiescence) [3]. AGA is characterized by a gradually decreased anagen phase; on the contrary, the duration of telogen remains unchanged or is protracted. Two main factors are involved in AGA etiology: androgens, such as testosterone and dihydrotestosterone (DHT), and genetic actors, which are able to affect the hair follicle response to circulating androgens [4,5]. In addition, the sustained microscopic follicular inflammation and free radicals, such as lipid peroxides, can also contribute to its onset affecting hair loss [6,7].

In hair follicles, testosterone is converted to DHT by the enzyme 5α-reductase. DHT is able to bind the androgen receptor (AR) in dermal papilla cells (DPCs) with a higher affinity compared to testosterone and leading to a prolonged telogen phase [8].

In order to treat AGA, a possible approach is to prolong the anagen phase using compounds able to exert an antioxidant and anti-inflammatory activity and/or to inhibit 5α-reductase reducing the androgenic effect.

Currently, topical minoxidil and oral finasteride represent the only two drugs approved by FDA (Food and Drug Administration) and EMA (European Medicines Agency) for the treatment of androgenetic alopecia [9]. The first one is a potassium channel opener, which promotes the proliferation and differentiation of DPCs, resulting in an extended anagen phase [10]. The second one is a 5α-reductase inhibitor able to prevent testosterone conversion into DHT [11]. However, these two therapeutic agents cause several adverse effects such as local irritation, bloating, erythema, tachycardia, myocardial infarction in the case of minoxidil and abnormal sexual function, confusion, cold sweats, faintness, dizziness, severe myopathy [1,12,13,14,15].

In addition to these conventional therapies, other treatments involve platelet-rich plasma (PRP) and adipose-derived stem cells (ASCs). PRP consists of a high concentration of platelets in a small volume of plasma containing several growth factors, such as platelet-derived growth factor (PDGF), vascular endothelial growth factor (VEGF), epidermal growth factor (EGF), and basic fibroblast growth factor (bFGF), which are released after platelet activation [16]. PRP promotes wound healing, angiogenesis, and tissue remodeling. Furthermore, several studies highlighted its beneficial effects in the treatment of AGA due to the ability to stimulate proliferation and differentiation of dermal papillar cells leading to an improvement of hair growth and density [17,18]. Adipose-derived stem cells can also find application in regenerative medicine. The pathways regulating their adipogenic differentiation involve the activity of receptor tyrosine kinases (RTKs), including fibroblast growth factor, vascular endothelial growth factor, ErbB receptors and the downstream-regulated serine/threonine protein kinase B (Akt) [19]. In particular, ASCs are able to stimulate hair growth, increasing the proliferation rate of human follicular cells, and to protect human dermal papilla cells against cytotoxic injury due to androgen and reactive oxygen species [20].

Recently, low-level laser therapy (LLLT) has been also proposed for the treatment of hair loss conditions such as androgenetic alopecia [21,22,23]. Several clinical trials, indeed, established that LLLT is able to stimulate hair growth in both men and women with relevant improvements and it may be used independently or in association with drugs such as minoxidil and finasteride. The main hypothesized mechanism of action involves the stimulation of epidermal stem cells in the hair follicle bulge and the shifting of the follicles into the anagen phase. Anyway, further studies need to be carried out in the aim to better understand LLLT mechanism of action and to identify the optimal power and wavelength.

Based on these considerations, natural products able to promote hair growth are receiving considerable attention, due to their safety, in the hair care field for the development of innovative and safe anti-baldness products.

In the present research study, *Origanum vulgare* leaf extract, *Camellia Sinensis* leaf extract, and *Capsicum Annuum* fruit extract were employed as sources of bioactive compounds to prepare an innovative anti-baldness agent, IPSTiC patch hair, based on a polymeric blend consisting of high molecular weight hyaluronic acid and soybean proteins.

*Origanum vulgare* (Lamiaceae) is a native plant present in Europe, Asia, America and North Africa [24]. The extracts prepared starting from its leaves are rich in many bioactive phenolic compounds, including rosmarinic acid, caffeic acid, and several flavonoids [25,26], which are responsible for the relevant antioxidant and anti-inflammatory activities [27,28,29,30,31,32]. These significant properties, which are useful in AGA management, explain *Origanum vulgare* health effects and, therefore, its application in traditional medicine.

*Camellia Sinensis* is an evergreen tree or shrub member of theaceae family. Tea leaves contain several bioactive molecules including polyphenols such as catechins and flavonoids, alkaloids such as caffeine, and vitamins. In green tea, the major constituent is represent by epigallocatechin-3-gallate (EGCG) [33,34,35]. Several studies highlighted the antioxidant and anti-inflammatory properties of tea polyphenols, which also present stress inhibitory effects [36,37,38]. In a work of Esfandiari et al. the authors investigated the effectiveness of tea polyphenols on hair loss in mice concluding that the anti-inflammatory activity and the stress inhibitory properties of these natural compounds affect hair regrowth [39]. Therefore, *Camellia Sinensis* exerts a significant hair growth promoting activity mainly due to its phytoconstituent epigallocatechin 3-gallate, which acts via proliferative and anti-apoptotic effects on dermal papilla cells and is able to inhibit 5α-reductase [39,40,41,42,43].

*Capsicum Annuum* L. is a domesticated species of the plant genus Capsicum belonging to the family of Solanaceae. The pigments responsible for the color of peppers are carotenoids, which are characterized by antioxidant and anti-inflammatory properties due to their ability to scavenge radical oxygen species (ROS) and reactive nitrogen species (RNS) [44,45,46,47]. The main bioactive compounds are capsaicin and isoflavone. In a research study, it was observed that the combined administration of these two molecules might increase insulin-like growth factor-I (IGF-I) production in hair follicles promoting hair growth [48]. Moreover, intradermal injection of capsaicin caused anagen induction in mice [49].

Given these characteristics, the aim of the present research study was the development of an innovative anti-baldness agent, which combines the hair growth promoting activities of bioactive ingredients, such as *Origanum vulgare* leaf extract, *Camellia Sinensis* leaf extract, and *Capsicum Annuum* fruit extract, with the higher stability and long-lasting effect due to the presence of a polymeric blend consisting of high molecular weight hyaluronic acid and soybean proteins.

## 2. Results and Discussion

### 2.1. Scavenging Effect on NO· Radical: Anti-Inflammatory Properties

The anti-inflammatory properties of IPSTiC patch hair was investigated by evaluating its ability to scavenge NO radicals, which are potent pro-inflammatory mediators [50].

The scavenging activity has been expressed as percent inhibition of the NO radical and calculated according to the following Equation (1):

(1) $$Inhibiton\;\left(\%\right)=\frac{A_{0}-A_{1}}{A_{0}}\times 100$$

where *A*_0_ is the absorbance of a control prepared in the same experimental conditions without the tested item, and *A*_1_ is the absorbance of the sample.

In the adopted experimental conditions, the tested item was able to inhibit NO· radicals with a percent inhibition equal to 72.4 ± 0.6%.

### 2.2. Determination of 5α-Reductase Inhibition Activity

The conversion of testosterone into the more active compound dihydrotestosterone (DHT), which is responsible for a prolonged telogen phase, is due to the activity of 5α-reductase (5AR).

The inhibition of this enzyme represents an effective strategy in the AGA management; therefore, the ability of the developed anti-baldness agent to inhibit 5AR was investigated using a homogenate from LNCaP cells as source of the enzyme.

The obtained results highlighted an inhibition percentage equal to 68.3 ± 0.7% for IPSTiC Patch Hair.

### 2.3. In Vitro Diffusion Studies: Long-Lasting Efficacy

The “long-lasting” efficacy of the developed IPSTiC patch hair was explored by carrying out in vitro diffusion studies using Franz diffusion cells.

For this purpose, Strat-M^®^ membranes were used as a synthetic alternative to human skin allowing to obtain data strictly related to the diffusion processes occurring through human skin.

**Figure 1:**
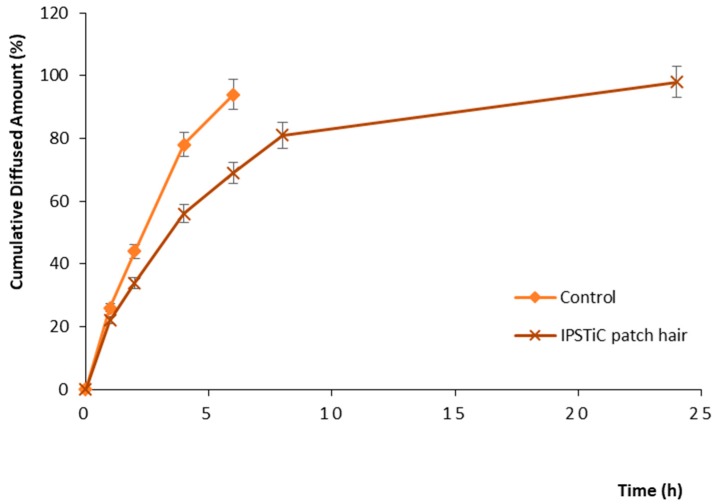
In vitro diffusion profiles.

The results were expressed as cumulative diffused amount (%) and the obtained diffusion profiles for each tested sample were reported in Figure 1.

The control sample consisting of a mixture of the starting bioactive ingredients, such as *Origanum vulgare* leaf extract, *Camellia Sinensis* leaf extract, and *Capsicum Annuum* fruit extract in the absence of the polymeric blend, showed a diffused amount equal to 94% within the first 6 h. On the other hand, IPSTiC patch hair exhibited at the same time a value of 69%, reaching 98% in 24 h. Therefore, the obtained profiles highlighted a protracted diffusion process for the developed anti-baldness agent.

### 2.4. Stability Studies

The present studies aim to investigate the stability of IPSTiC patch hair under different degradation conditions.

For this purpose, all the performed tests were carried out on the developed anti-baldness agent and on a control sample consisting of a mixture of the starting bioactive ingredients, such as *Origanum vulgare* leaf extract, *Camellia Sinensis* leaf extract, and *Capsicum Annuum* fruit extract, in the absence of the polymeric blend.

#### 2.4.1. Thermal Stability under Stress Conditions

The samples were kept at 4 °C for three days, then at 25 °C for the successive three days and, finally, heated at 50 °C for the last three days. The samples were stored in dark conditions for the duration of the test and stability was assessed at each temperature point.

**Figure 2:**
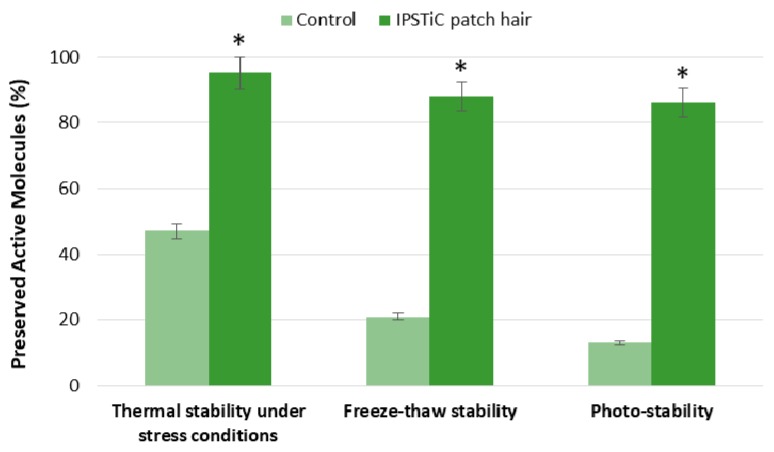
Percentage of preserved active molecules for control sample and IPSTiC patch hair after the stability studies performed under different conditions. Asterisks denote values that were significantly different from the vehicle control (*p* < 0.05).

Both the tested items were found to be stable at 4 °C and 25 °C; on the contrary, exposure to higher temperatures, such as 50 °C, resulted in a degradation of the active ingredients contained in the control equal to 53%. This degradation was not observed in the case of IPSTiC patch hair (Figure 2).

#### 2.4.2. Freeze–Thaw Stability

The samples were kept at 2–8 °C for 2 days and, then, heated at 40 °C for 2 days per cycle; three cycles of freeze–thaw were conducted. The freeze–thaw stability was evaluated after the third cycle and the obtained results were reported in Figure 2.

At the end of the third cycle, a degradation of 79% was observed for the control sample; on the contrary, the observed value for IPSTiC patch hair was only 12%.

#### 2.4.3. Photo-Stability

The photo-stability was investigated by treating the control sample and the anti-baldness agent under strong light at room temperature for 5 days.

On the fifth day of treatment, almost all the active ingredients contained in the control underwent degradation; on the other hand, a percentage equal to 86% was preserved by the IPSTiC system (Figure 2).

The results obtained from the performed studies confirmed the significant ability of the polymeric blend to improve the stability of the active ingredients, both in the case of thermal stress and in the presence of light. Therefore, the developed IPSTiC technology allows the prevention of thermal degradation and photolysis of the bioactive compounds contained in *Origanum vulgare* leaf extract, *Camellia Sinensis* leaf extract, and *Capsicum Annuum* fruit extract, increasing their stability and preserving their properties.

### 2.5. Safety Assessment

#### 2.5.1. Skin Irritation

In order to evaluate the skin irritation potential of IPSTiC patch hair, the EPISKIN prediction model was employed according to OECD TG439 version 2015.

**Figure 3:**
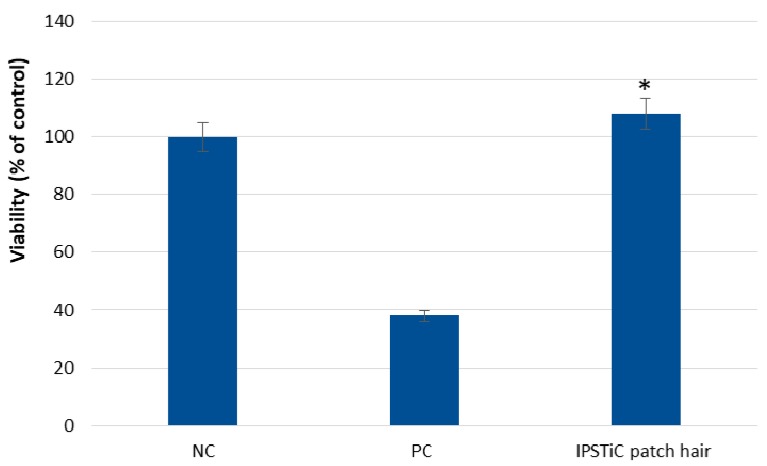
Effect of IPSTiC patch hair in EPISKIN™ RHE/L/13. Asterisks denote values that were significantly different from the vehicle control (*p* < 0.05).

The in vitro reconstructed human epidermis was treated with the developed anti-baldness agent at a concentration of 16 μL ± 0.5 μL using a nylon mesh according to the “42 bis” procedure. This experimental protocol consists of a 42 min exposure followed by a rinsing step and a 42 h post-incubation. After the incubation, cell viability was assessed by the MTT assay and the obtained data were reported in Figure 3.

The developed anti-baldness agent did not exhibit irritation effects in EPISKIN™ RHE/L/13 human skin equivalent. On the contrary, the positive control induced a significant reduction in cell viability.

#### 2.5.2. In Vitro Analysis of the Pro-Sensitising Potential

The human Cell Line Activation Test (h-CLAT) aims to evaluate if a product is a skin sensitiser or non-sensitiser. For this purpose, THP-1 cell line was employed and the expression of CD54 and CD86 was investigated. The increasing level of expression of these two co-stimulatory molecules on monocytes, indeed, represents a signal of activation of the immune response due to the exposure to potentially sensitising substances.

Firstly, the propidium iodure (PI) uptake assay was carried out in order to identify the highest concentration that does not cause cell mortality higher than 25%. Cell viability was calculated using the following Equation (2):

(2) $$\mathrm{Cell}\;\mathrm{Viability}=\left(\frac{\mathrm{Number}\;\mathrm{of}\;\mathrm{living}\;\mathrm{cells}}{\mathrm{Total}\;\mathrm{number}\;\mathrm{of}\;\mathrm{acquired}\;\mathrm{cells}}\right)\times 100$$

The *CV75* value, which represents the concentration showing 75% of THP-1 cell survival, was calculated by log-linear interpolation according to the following Equation (3):

(3) $$Log\;CV75=\frac{\left(75-B\right)\times Log\left(C\right)-\left(75-A\right)\times Log\left(D\right)}{A-B}$$

where *A* is cell viability >75%; *B* is cell viability <75%; and *C* or *D* is the concentration showing the value of cell viability *A* or *B*. The obtained *CV75* value was used to determine the highest concentration of tested item to be used in the final test.

Then, cells were treated with IPSTiC patch hair and the expression of CD86 and CD54 was analyzed by flow cytometry. Based on the geometric mean fluorescence intensity (MFI), which is proportional to the expression of co-stimulatory molecules, the relative fluorescence intensity (RFI) of CD86 and CD54 for positive control cells and treated cells were calculated using the following Equation (4):

(4) $$\mathrm{RFI}=\frac{\mathrm{MFI}\;\mathrm{of}\;\mathrm{treated}\;\mathrm{cells}-\mathrm{MFI}\;\mathrm{of}\;\mathrm{treated}\;\mathrm{isotype}\;\mathrm{cells}}{\mathrm{MFI}\;\mathrm{of}\;\mathrm{solvent}\;\mathrm{treated}\;\mathrm{control}\;\mathrm{cells}-\mathrm{MFI}\;\mathrm{of}\;\mathrm{solvent}\;\mathrm{treated}\;\mathrm{isotype}\;\mathrm{control}\;\mathrm{cells}}$$

If the RFI % value of CD86 is equal to or greater than 150% at any tested dose (>50% of cell viability) in at least two independent runs and/or if the RFI of CD54 is equal to or greater than 200% at any tested dose (>50% of cell viability) in at least two independent runs, the sensitization prediction is considered as positive. Otherwise, it is considered as negative.

**Figure 4:**
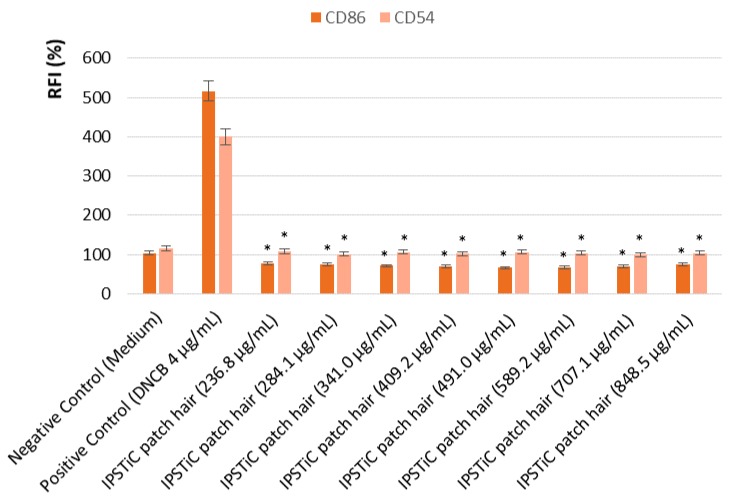
Expression of CD86 and CD54. Asterisks denote values that were significantly different from the vehicle control (*p* < 0.05).

The obtained results, reported in Figure 4, confirmed that the developed product IPSTiC patch hair did not show any sensitising potential.

## 3. Materials and Methods

### 3.1. Chemicals

Reagents were purchased from Sigma-Aldrich (Milan, Italy).

The polymeric blend IPSTiC patch hair, consisting of high molecular weight hyaluronic acid and soybean proteins and containing bioactive ingredients such as *Origanum vulgare* leaf extract, *Camellia Sinensis* leaf extract, and *Capsicum Annuum* fruit extract, was supplied by Macrofarm s.r.l. The extracts used to formulate the tested product IPSTiC patch hair were prepared starting from the same batch and all the performed experiments were carried out in triplicate on the same production lot. For this purpose, *Origanum vulgare* leaves, *Camellia Sinensis* leaves and *Capsicum Annuum* fruits were extracted using a water/glycerol mixture as extraction solvent under specific temperature and time conditions.

The EPISKIN™ RHE/L/13 human skin equivalent kit was obtained from SkinEthic Laboratories (Lyon, France).

All solvents were reagent or HPLC grade and purchased from VWR (Milan, Italy).

### 3.2. Cell Cultures

Androgen-dependent prostate cancer cells (LNCaP) were obtained from the American Type Culture Collection (ATCC, CRL-1740™) and used as source of the enzyme 5α-reductase (5AR). LNCaP cells were cultured in RPMI-1640 medium supplemented with 10% (*v*/*v*) fetal bovine serum and 100 U/mL penicillin G and 100 µg/mL streptomycin at 37 °C in a 5% CO_2_ humidified atmosphere.

Human monocytic leukemia cell line THP-1 from ATCC was employed for the in vitro evaluation of the pro-sensitising potential. Cells were kept in RPMI containing 10% fetal bovine serum (FBS), 0.05 mM 2-mercaptoethanol, 100 units/mL penicillin, and 100 µg/mL streptomycin.

### 3.3. Instrumentation

Absorption spectra were recorded with a Jasco V-530 UV/Vis spectrometer (Jasco Europe S.R.L., Cremella (LC), Italy).

The HPLC analyses were carried out using a Jasco PU-2080 liquid chromatograph (Jasco Europe S.R.L., Cremella (LC), Italy) equipped with a Rheodyne 7725i injector (fitted with a 20-µL loop), a Jasco UV-2075 HPLC detector, a Jasco-Borwin integrator, and a 250 × 4.6 mm C18 Luna^®^ column, 5 µm particle size (Phenomenex, Torrance, CA, USA). The adopted mobile phase was a mixture of methanol/water (80:20) at a flow rate of 0.5 mL/min and the UV detection wavelength was 242 nm.

In vitro diffusion studies were carried out employing Franz diffusion cells (Disa, Milan, Italy; permeation area 0.4614 cm^2^).

### 3.4. Scavenging Effect on NO· Radical

In order to investigate the anti-inflammatory properties of IPSTiC patch hair, its ability to scavenge NO· radicals was evaluated using sodium nitroprusside (SNP) as a source of radicals according to the experimental protocol reported in literature with slight modifications [51].

A 5 mM solution of SNP was prepared in phosphate-buffered saline (PBS) at pH 7.3. Then, 5 mL of this solution were mixed with 0.5 mL of the tested anti-baldness agent and incubated for 180 min at 25 °C in front of a visible polychromatic light source (25 W tungsten lamp). The produced NO· radicals interact with oxygen leading to the formation of nitrite ion (NO_2_^−^), which was determined by adding 1 mL of Griess reagent to 1 mL of the incubation mixture and measuring the absorbance at 546 nm.

The assay was repeated in triplicate and data were expressed as mean values (±SD).

### 3.5. Determination of 5a-Reductase Inhibition Activity

In the aim to evaluate the ability of IPSTiC patch hair to inhibit 5a-reductase (5AR), the homogenate from LNCaP cells, prepared following the procedure reported in literature [15], was used as source of the enzyme.

The prepared reaction mixture consists of 0.2 mL of PBS (pH 7.2), 0.7 mL of homogenate, 0.1 mL of testosterone (2.88 mg/mL), and 0.3 mL of the tested item. The reaction was started by adding 0.2 mL of NADPH-Na_4_ (2 mg/mL) and, after 1 h at 37 °C, it was stopped by the addition of 3 mL of ethyl acetate. The samples were shaken for 1 min and, then, centrifuged at 3500 rpm for 15 min. The ethyl acetate layer was evaporated and the obtained residue was dissolved in 2 mL of methanol in order to detect the residual testosterone content by HPLC analysis [52].

The ability to inhibit 5α-reductase has been expressed as percentage according to the following Equation (5):

(5) $$\mathrm{Inhibiton}\;\left(\%\right)=\frac{C_{0}-C_{1}}{C_{0}}\times 100$$

where *C* is the amount of testosterone converted into dihydrotestosterone (DHT) by 5AR (*C* = testosterone_0min_ − amount of testosterone_60min_), *C*_0_ is the amount of converted testosterone of a control prepared in the same experimental conditions without the tested item, and *C*_1_ is the amount of converted testosterone of the sample.

The protocol was performed in triplicate and data were expressed as mean values (±SD).

### 3.6. In Vitro Diffusion Studies by Vertical Franz Cells

The long-lasting efficacy of IPSTiC patch hair was investigated by performing in vitro diffusion studies using Franz diffusion cells according to the experimental procedure already reported in literature [53].

The same experimental conditions were applied to a control sample consisting of a mixture of the starting bioactive ingredients, such as *Origanum vulgare* leaf extract, *Camellia Sinensis* leaf extract, and *Capsicum Annuum* fruit extract, without the polymeric blend.

The in vitro diffusion studies were repeated in triplicate and the obtained results were expressed as diffused amount (%) of the tested items.

### 3.7. Stability Studies

#### 3.7.1. Thermal Stability under Stress Conditions

The thermal stability of IPSTiC patch hair was investigated by keeping the sample at 4 °C, 25 °C, and 50 °C for three days for each temperature [54].

Stability was assessed at each temperature point by spectrophotometric analysis and the obtained results were expressed as percentage of preserved active molecules.

The same conditions were applied on a control sample consisting of a mixture of the starting bioactive ingredients, such as *Origanum vulgare* leaf extract, *Camellia Sinensis* leaf extract, and *Capsicum Annuum* fruit extract, in the absence of the polymeric blend.

The experiments were carried out in triplicate.

#### 3.7.2. Freeze–Thaw Stability

In order to investigate the stability of the developed anti-baldness agent, IPSTiC patch hair was subjected to three freeze–thaw cycles involving 2 days at 2–8 °C and 2 days at 40 °C per cycle [54].

The freeze–thaw stability was evaluated after the third cycle by spectrophotometric analysis and the obtained results were expressed as percentage of preserved active molecules.

The same experimental conditions were applied on a control sample consisting of a mixture of the starting bioactive ingredients, such as *Origanum vulgare* leaf extract, *Camellia Sinensis* leaf extract, and *Capsicum Annuum* fruit extract, in the absence of the polymeric blend.

The experiments were carried out in triplicate.

#### 3.7.3. Photo-Stability

The developed product IPSTiC patch hair was exposed to strong light (4500 Lx ± 500 Lx), which was comparable to natural light, at room temperature for 5 days [54] and, then, the sample was analysed by using an UV/Vis spectrometer.

The same conditions were applied on the control sample and the experiments were performed in triplicate.

### 3.8. Safety Assessment

#### 3.8.1. Skin Irritation

In order to evaluate skin irritation of IPSTiC patch hair, the EPISKIN™ RHE/L/13 human skin was treated with a solution of the anti-baldness agent (16 μL ± 0.5 μL), PBS as a negative control and 5% (*w*/*v*) SDS as a positive control according to the protocol reported in the literature [51] and OECD TG439 version 2015.

Each sample was applied on triplicate tissues.

#### 3.8.2. In Vitro Analysis of the Pro-Sensitising Potential

The aim of the test is to evaluate the pro-sensitising potential of the prepared product. For this purpose, the human monocytic leukemia cell line THP-1 was treated with IPSTiC patch hair and the expression of two co-stimulatory molecules, such as CD54 and CD86, was checked out. The test was performed according to the method described in OECD 442E and in the 158 EURL-ECVAM protocol (European Union Reference Laboratory for alternatives to animal testing).

In order to assess the highest concentration, which does not cause cell mortality higher than 25%, a viability assay, such as the propidium iodure (PI) uptake, was carried out. For this purpose, cells were distributed into a 96-well flat-bottom plate with 1.6 × 10^5^ cell/s/well and, after 24 h, the culture medium was mixed 1:1 with the tested item and controls. The wipes were wrung out and the test was carried out on the liquid part. The liquid was dissolved in phosphate buffer and 8 serial dilutions were prepared to be tested on the cells. Culture medium was used as negative control. After 24 h of incubation, cells were collected by centrifugation and re-suspended with FACS buffer in the presence of iodure propidium (PI) for cytometry analysis. The CV75 value was calculated (concentration that caused 25% of cell mortality) and used as the highest concentration in the final test.

According to the obtained preliminary results, the tested item was diluted in phosphate buffer at the concentration corresponding to 100 times the 1.2 C CV75. Then 1:1.2 serial dilutions were made in order to prepare eight stock solutions, ranging from 0.335 × CV75 to 1.2 × CV75, to be tested. The prepared stock solutions were further diluted 1:50 into culture medium and these final solutions were used for treatment with a further 1:2 dilution factor. Culture medium and 2,4-dinitrochlorobenzene (DNCB, 4 µg/mL) were employed as negative and positive controls, respectively. The treatment involved an exposure of 24 h at 37 °C and with 5% CO_2_. Then, cells were collected by centrifugation, re-suspended in FACS buffer and divided into three aliquots. After centrifugation, cells were re-suspended in the blocking solution (FACS buffer containing 0.01% of human gamma-globulins) and incubated at 4 °C for 15 min. After centrifugation, cells were incubated with a fluoresceinated anti-CD86, anti-CD54 or mouse IgG1 (control isotype) antibodies for 30 min at 4 °C. Finally, after washing with FACS buffer, cells were re-suspended in FACS buffer in the presence of PI solution and the expression levels of CD86 and CD54 and cell viability were evaluated by flow cytometry.

### 3.9. Statistical Analysis

All the tests were performed in triplicate. All data were expressed as mean ± SD and Student’s t test was used. *p* values < 0.05 were considered statistically significant.

## 4. Conclusions

In the present study, a novel anti-baldness agent, based on an effective strategy for the delivery of bioactive molecules called IPSTiC (Interconnected PolymerS TeChnology), was developed.

The obtained product IPSTiC patch hair consists of a polymeric blend made up of high molecular weight hyaluronic acid and soybean proteins that is able to increase the efficacy and stability of bioactive ingredients such as *Origanum vulgare* leaf extract, *Camellia Sinensis* leaf extract, and *Capsicum Annuum* fruit extract. Soybean proteins and hyaluronic acid chains, indeed, interact by interactions, which are similar to the biological ones occurring between polysaccharides and proteins, leading to the formation of a network. This polymeric blend, which is able to stabilize and release in a prolonged way the active compounds contained in the extracts, is at the base of the developed IPSTiC technology.

IPSTiC patch hair was investigated for its anti-hair loss properties. Several mechanisms are involved in this process and, in this work, the attention was focused on 5α-reductase and NO radicals. The ability to inhibit 5α-reductase activity and NO radicals, which are involved in inflammatory processes, was observed confirming the efficacy of the developed anti-baldness agent. Moreover, the adopted IPSTiC technology allowed the improvement of the stability of the bioactive ingredients under different stress conditions, and reaching a long-lasting release.

Finally, the biocompatibility of IPSTiC patch hair was also assessed by both in vitro analysis of the pro-sensitising potential and EPISKIN model.

The obtained results in terms of efficacy and safety support the potential use of this product in the topical treatment of AGA.
